# The prognostic significance of lung immune prognostic index in patients with osteosarcoma after chemotherapy

**DOI:** 10.3389/fonc.2025.1561343

**Published:** 2025-09-29

**Authors:** Han Liu, Hui Kang, Jianhua Mu, Jingjing Wang, Taojun Gong, Zhuangzhuang Li, Xuanhong He, Yuqi Zhang, Li Min, Minxun Lu, Chongqi Tu

**Affiliations:** ^1^ Department of Orthopedics, Orthopaedic Research Institute, West China Hospital, Sichuan University, Chengdu, China; ^2^ Department of Operating Room, West China Hospital, Sichuan University/Nursing Key Laboratory of Sichuan, Sichuan University, Chengdu, China; ^3^ Department of Endocrine, Sichuan Provincial People’s Hospital, School of Medicine,University of Electronic Science and Technology of China, Chengdu, China

**Keywords:** osteosarcoma, lipi, prognostic nomograms, chemotherapy, prediction model

## Abstract

**Background:**

Osteosarcoma is the most common primary malignant bone tumor. However, research on predicting the prognosis of patients with osteosarcoma after chemotherapy (POC) remains limited. Notably, the Lung Immune Prognostic Index (LIPI) has emerged as a novel and effective prognostic factor in lung cancer. Therefore, this study aims to explore the prognostic significance of LIPI in POC for the first time, providing new insights and a foundation for evaluating the prognosis of these patients.

**Methods:**

This retrospective study analyzed patients with POC who were admitted to our center between January 2012 and January 2022. Hematological and clinical characteristics were collected and systematically evaluated. Kaplan–Meier survival analysis and Cox regression models were employed to assess the associations between various prognostic factors and overall survival (OS). Independent risk factors influencing OS were identified through both univariate and multivariate analyses. Based on these findings, a LIPI nomogram model was developed to predict OS in patients with POC.

**Results:**

This study included 150 patients who underwent chemotherapy, with 41 (27%), 80 (53%), and 29 (19.3%) classified into poor, moderate, and good prognostic categories, respectively, based on the LIPI classification (P < 0.0001). Time-dependent receiver operating characteristic (ROC) curve analysis demonstrated that LIPI exhibited superior prognostic predictive capability compared to other hematological and clinical parameters. Univariate and multivariate analyses identified LIPI as an independent prognostic factor. A nomogram was subsequently developed by integrating significant prognostic variables. Calibration curves confirmed the nomogram’s accuracy in predicting three- and five-year overall survival (OS) post-chemotherapy. Furthermore, decision curve analysis indicated that the LIPI-based nomogram would provide substantial clinical benefits for chemotherapy patients.

**Conclusion:**

This study assessed the prognostic efficacy of LIPI in patients with POC and developed a LIPI-based nomogram to assist clinicians in predicting three- and five-year overall survival (OS). The proposed model has the potential to facilitate timely interventions and guide personalized management strategies, thereby improving patient outcomes.

## 1 Introduction

Osteosarcoma is the most common primary malignant bone tumor, primarily affecting adolescents and the elderly. The current standard treatment includes radical resection and neoadjuvant chemotherapy (1, 2). With the introduction of chemotherapy in cancer treatment, the 5-year OS rate has improved to 50%–70% (3). However, outcomes for osteosarcoma patients remain poor due to drug resistance, distant metastasis, and/or local recurrence (4). Therefore, identifying significant prognostic factors for osteosarcoma is urgently needed. Previous studies have highlighted the prognostic value of several biomarkers in osteosarcoma, each with its advantages and limitations. Traditional prognostic factors, such as Enneking stage, tumor size, metastasis, and pathological fractures, are helpful in guiding treatment decisions but are limited in their prognostic power, as they focus on only a single aspect of clinical or pathological features (5). New prognostic factors, including microRNAs, long non-coding RNAs, and gene signatures, have shown promise in predicting patient outcomes. However, the high costs and practical limitations of these novel factors hinder their widespread clinical application (6–10). As a result, a simple, accurate, and cost-effective prognostic factor for osteosarcoma is urgently needed to improve patient outcomes in POC.

Tumor-associated inflammation plays a critical role in tumor progression (11–13). Several inflammation-related markers, including the neutrophil-to-lymphocyte ratio (NLR), platelet-to-lymphocyte ratio (PLR), lymphocyte-to-monocyte ratio (LMR), and serum lactate dehydrogenase (LDH), have been shown to effectively predict the OS of patients with lung cancer, gastric cancer, and pancreatic ductal adenocarcinoma (14–18). The LIPI, calculated using the baseline-derived neutrophils/(leukocytes minus neutrophils) ratio (dNLR) and serum LDH, has proven to be a valid prognostic indicator for malignancies treated with immune checkpoint inhibitors or chemotherapy (19–21). Furthermore, LIPI and related predictive models have also been explored for osteosarcoma (22). However, to our knowledge, the utility of LIPI in predicting the prognosis of POC remains unclear.

In this retrospective study, we aim to explore the prognostic significance of LIPI in predicting outcomes for POC. Additionally, we developed a LIPI-based prognostic nomogram for POC.

## 2 Patients and methods

### 2.1 Patients

**Figure 1:**
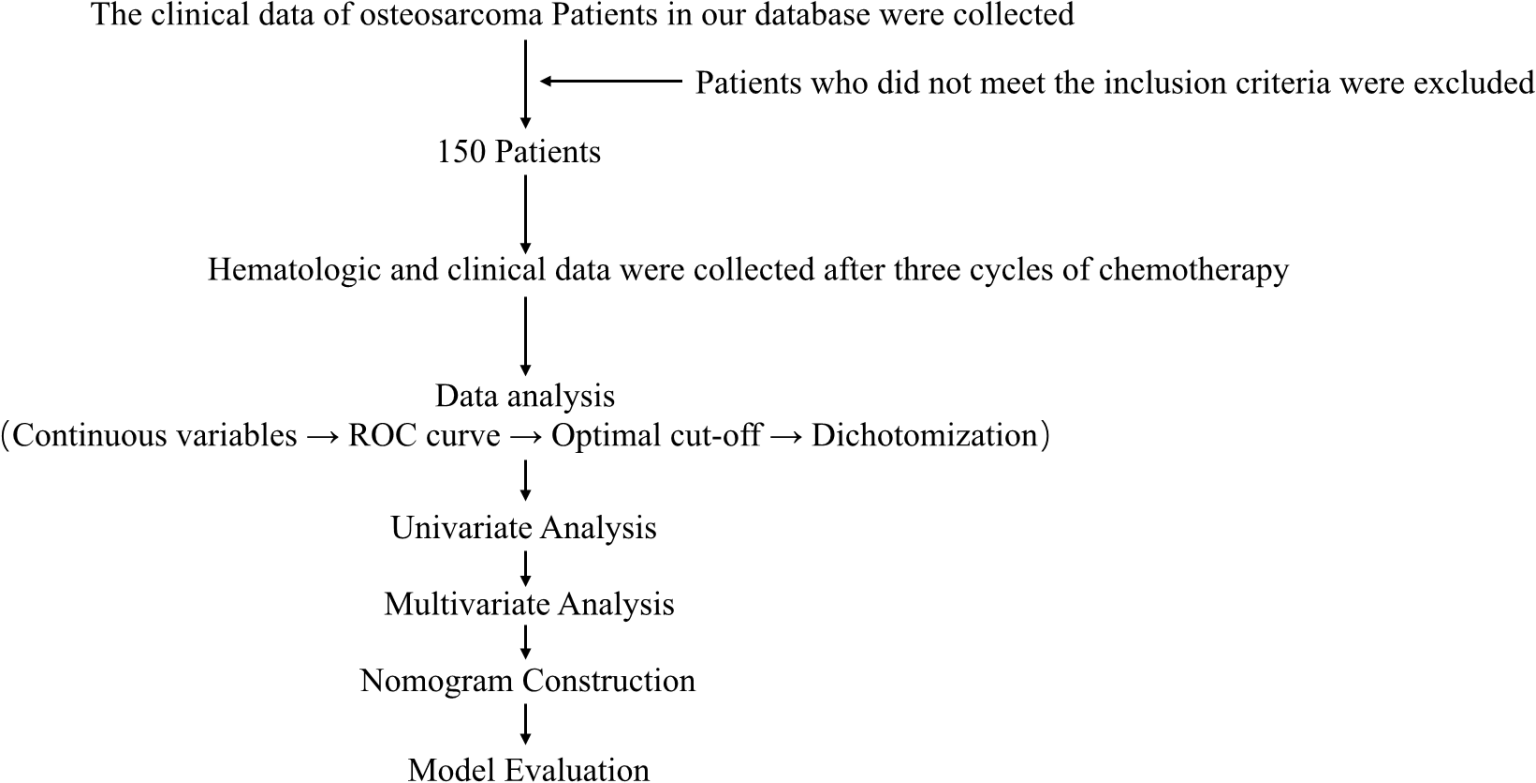
Work flow chart of this study.

The flow chart through this study is presented in **Figure 1**. With approval from the Medical Ethics Committee, we retrospectively reviewed the clinical data of osteosarcoma patients recorded between January 2012 and January 2022 in the database of the Musculoskeletal Tumor Center at West China Hospital. Patient selection was conducted based on the following inclusion criteria: (1) histopathologically confirmed high-grade osteosarcoma; (2) availability of complete hematological test results following neoadjuvant chemotherapy; and (3) administration of three cycles of neoadjuvant chemotherapy at our institution prior to surgery. The exclusion criteria were as follows: (1) histopathologically confirmed low-grade osteosarcoma (intramedullary and bone surface) or periosteal osteosarcoma; (2) prior neoadjuvant chemotherapy received before the first consultation at our hospital; (3) presence of hematological disorders; (4) diagnosis of other malignancies; and (5) failure to receive standard treatment, including cases of misdiagnosis, mistreatment, or incomplete postoperative chemotherapy. After applying these criteria, a total of 150 patients were included in the study. Each patient was followed up regularly until death or until January 2022. The follow-up schedule adhered to the following protocol: reexaminations every 3 months within the first year post-surgery, every 4 months during years 1–2, every 5 months during years 2–3, every 6 months during years 3–5, and annually beyond 5 years post-surgery.

### 2.2 Data collection and analysis

Hematological markers, including leukocyte count (Leut#), neutrophil count (Neut#), lymphocyte count (LYMPH#), platelet count (PLT), and lactate dehydrogenase (LDH), were obtained from the initial blood tests of patients with POC. The neutrophil-to-lymphocyte ratio (NLR), platelet-to-lymphocyte ratio (PLR), and derived neutrophil-to-lymphocyte ratio (dNLR) were calculated using the following formulas: NLR = Neut#/LYMPH#, PLR = PLT/LYMPH#, and dNLR = Neut#/(Leut# - Neut#).

In addition, clinical variables, including age, gender, and tumor location, were collected and analyzed. Overall survival (OS) was defined as the time from the date of diagnosis to the date of death or the last follow-up. The receiver operating characteristic (ROC) curve was used to determine the optimal cutoff values for each hematological index, which were subsequently dichotomized into binary variables for further analysis.

### 2.3 Establishment and validation of the LIPI in POC

The derived neutrophil-to-lymphocyte ratio (dNLR) was combined with lactate dehydrogenase (LDH) to construct the LIPI for POC. The prognostic impact of LIPI, along with clinical characteristics and other hematological variables, on overall survival (OS) in POC was then evaluated. To determine whether LIPI serves as an independent prognostic predictor, univariate and multivariate analyses were conducted. Variables identified as significant in the univariate analysis were subsequently included in the multivariate analysis to identify independent prognostic factors for POC.

### 2.4 Construction and evaluation of the LIPI-based nomogram for POC

Based on the aforementioned process, significant prognostic predictors were systematically selected and integrated to develop a nomogram. The total score for each patient was determined by summing the individual scores assigned to each prognostic factor. The nomogram visually represents the total score alongside the corresponding probability of overall survival (OS). To assess the discriminative ability and predictive accuracy of the nomogram, Harrell’s concordance index (C-index) and calibration curves were utilized, with the diagonal line serving as the reference for an optimal prediction model. Additionally, decision curve analysis (DCA) was conducted to evaluate the clinical utility of the nomogram by estimating net benefits across a range of threshold probabilities.

### 2.5 Statistical analysis

During data analysis, continuous variables were dichotomized according to the optimal cut-off values determined by receiver operating characteristic (ROC) curve analysis. Univariate Cox proportional hazards regression was then performed to evaluate the association between each variable and prognosis, with a significance level set at p < 0.05. Variables that reached statistical significance were subsequently entered into a multivariable Cox regression model to identify independent prognostic factors. A nomogram was constructed based on the regression coefficients of the multivariable model to provide individualized risk prediction. The clinical utility of the model was further assessed using decision curve analysis (DCA).

The Kolmogorov-Smirnov test was employed to assess the normality of continuous variables. Based on the normality test results, differences between continuous variables were analyzed using either the Mann-Whitney U test or Spearman correlation analysis. Categorical variables were evaluated using the chi-square test or Fisher’s exact test, depending on the sample size within each group. All statistical analyses were conducted using R software (version 4.1.0; Institute for Statistics and Mathematics, Vienna, Austria). A p-value of < 0.05 was considered indicative of statistical significance.

## 3 Results

### 3.1 Patient demographics and optimal cutoff values of hematological factors

**Figure 2:**
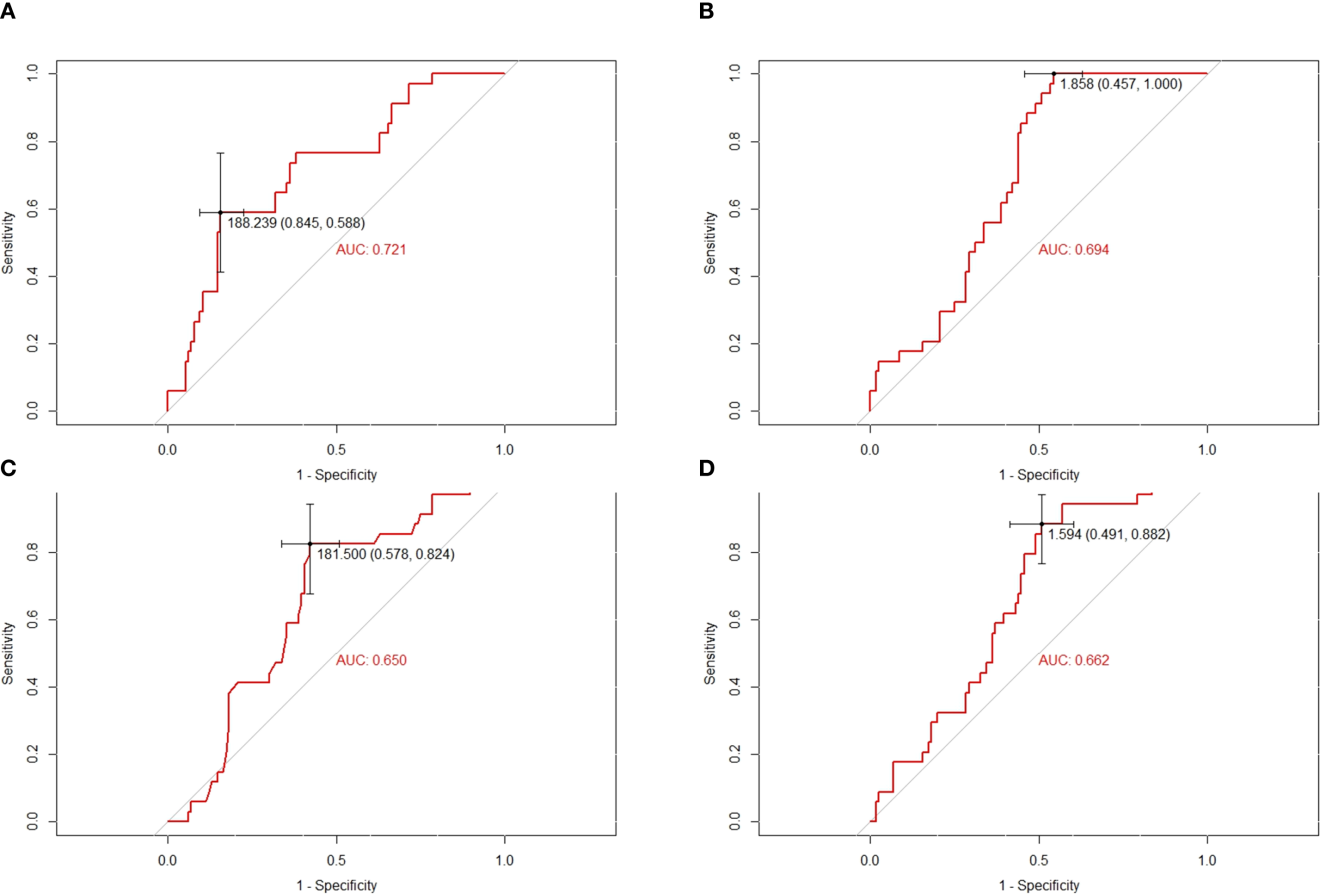
Conducting ROC analysis for various hematologic biomarkers. **(A–D)** The AUC and optimal cutoff values of PLR, NLR, LDH and dNLR are as follows. Sensitivity is represented on the vertical axis, while 1-specificity is depicted on the horizontal axis.

The baseline characteristics of the patients are summarized in **Table 1**. A total of 150 patients were included in this study, comprising 92 males and 58 females. The age of the patients ranged from 7 to 51 years, with a mean age of 20 years. Tumors were predominantly located in the extremities (94.0%), while 9 cases (6.0%) involved extra-extremity sites. The area under the curve (AUC) and optimal cutoff values for the platelet-to-lymphocyte ratio (PLR), neutrophil-to-lymphocyte ratio (NLR), lactate dehydrogenase (LDH), and derived neutrophil-to-lymphocyte ratio (dNLR) were determined. The respective AUCs and optimal cutoff values were as follows: 0.721 and 188.239 for PLR, 0.694 and 1.858 for NLR, 0.650 and 181.500 for LDH, and 0.662 and 1.594 for dNLR (**Figures 2A–D**).

**Table 1:** Clinical characteristics of patients.

	Patients	LIPI			P-value
		Poor	Middle	Good	
Total Patients	150	41	80	29	–
Age					0.012
>20	86	17	29	18	
≤20	64	24	51	11	
Sex					0.184
Male	92	18	55	19	
Female	58	23	25	10	
Tumor location					0.447
Extremities	141	39	75	27	
None-extremitis	9	2	5	2	
NLR					<0.001
>1.858	97	39	51	7	
≤1.858	53	2	29	22	
PLR					0.042
>188.239	38	9	23	9	
≤188.239	112	32	57	23	

### 3.2 Establishment and validation of the LIPI in POC

**Figure 3:**
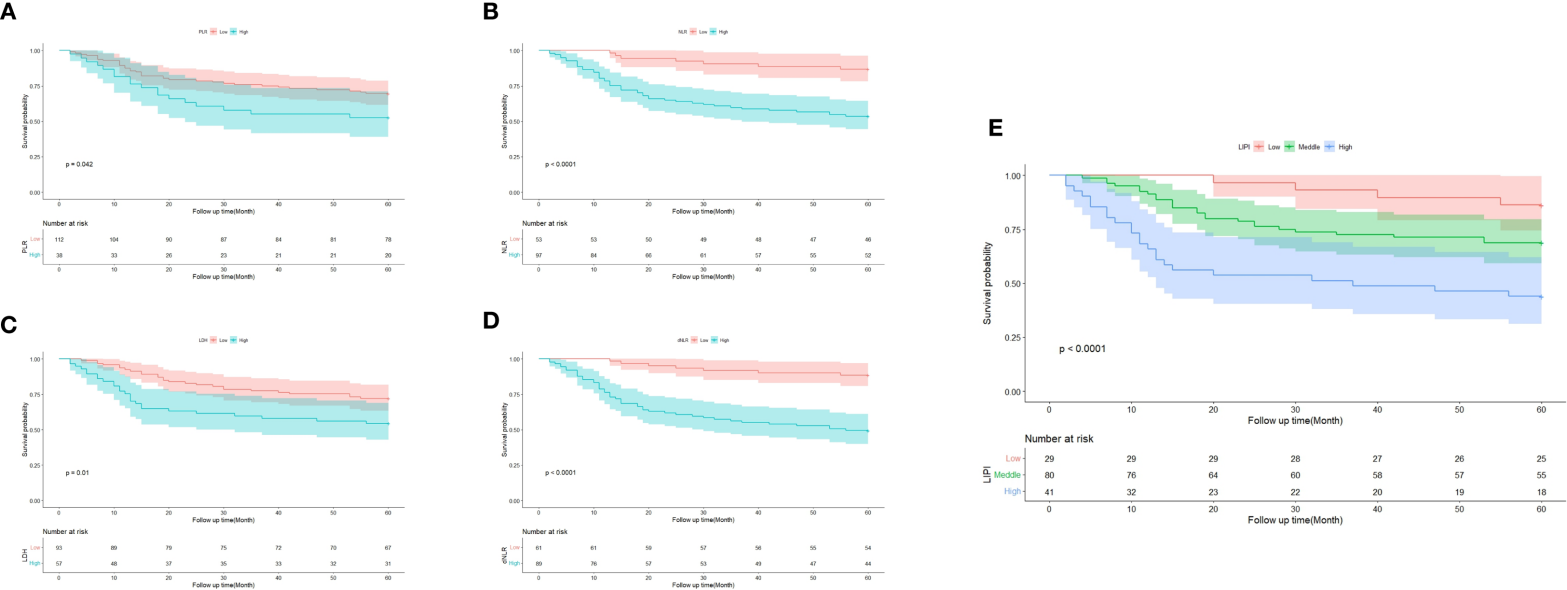
Predictive ability of different hematological biomarkers in POC. **(A–E)** Prognostic predictive effect of different inflammatory biomarkers in POC. Cumulative hazard function was plotted by the Kaplan-Meier methodology and the p value was calculated with two-sided log-rank tests. According to the logistic regression analysis, the differences between three LIPI groups in the survival probability were significant.

A total of 150 osteosarcoma patients who had undergone chemotherapy were stratified into different groups based on various hematological biomarkers. Patients in the low PLR group demonstrated a significantly better survival probability compared to those in the high PLR group (P = 0.042) (**Figure 3A**). Similarly, patients with a low NLR exhibited superior survival outcomes compared to those with a high NLR (P < 0.001) (**Figure 3B**). In addition, the low LDH group showed a significantly improved survival probability compared to the high LDH group (P = 0.010) (**Figure 3C**). Likewise, patients with a low dNLR had better survival outcomes than those with a high dNLR (P < 0.001) (**Figure 3D**).

Subsequently, the LIPI was constructed by integrating LDH and dNLR, following the method described by Mezquita et al (21). Based on LIPI classification, patients were stratified into three prognostic groups: 29 patients in the good LIPI group, 80 in the intermediate LIPI group, and 41 in the poor LIPI group (P < 0.0001) (**Figure 3E**). For instance, a patient with low dNLR and low LDH was categorized into the poor LIPI group, indicating an unfavorable survival prognosis.

**Figure 4:**
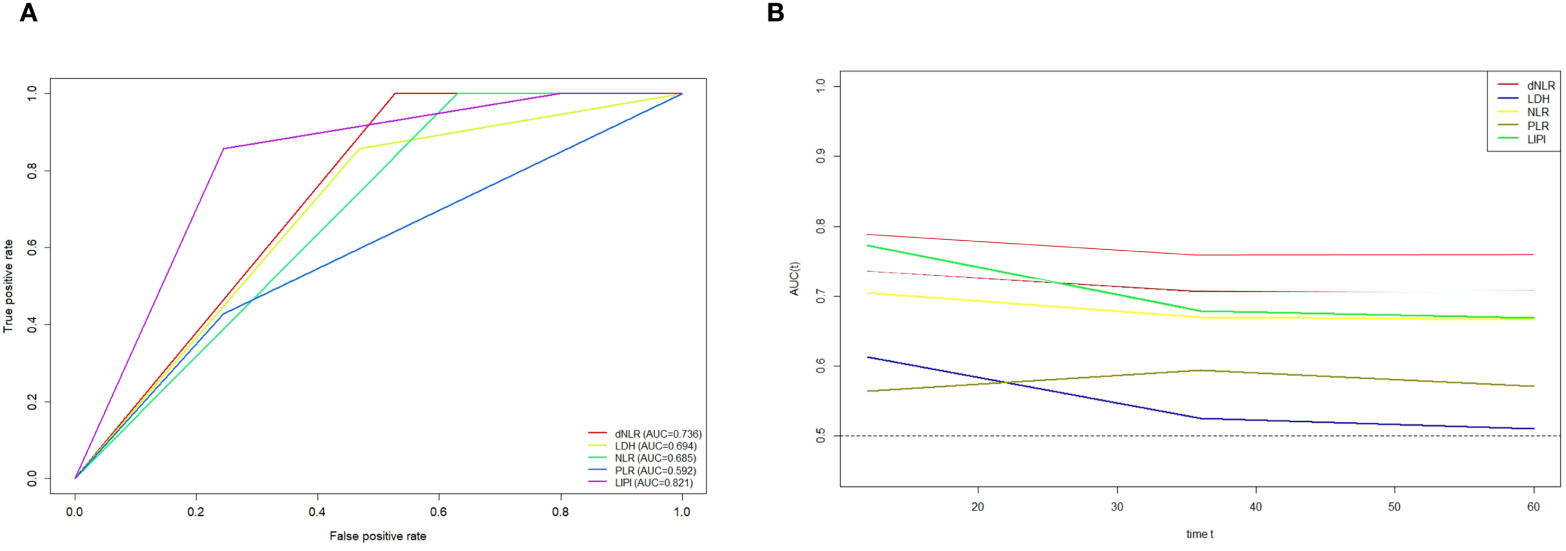
**(A)** ROC curves showing the predictive power of LIPI in POC versus a single hematology; **(B)** Time-dependent ROC curves illustrate the variances in predictive capabilities of different hematologic markers.

Furthermore, ROC curve analysis demonstrated that LIPI exhibited a markedly improved predictive ability compared to individual hematological markers (**Figure 4A**). The time-dependent ROC (t-ROC) curve further revealed that LIPI had a larger AUC than other individual hematological indices, including NLR, PLR, dNLR, and LDH, indicating its superior prognostic value (**Figure 4B**).

### 3.3 Univariate analysis and multivariate analysis

**Figure 5:**
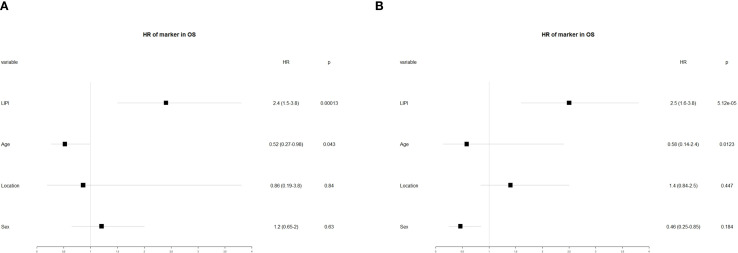
**(A)** Conducting univariate analysis for clinical characteristics and hematological biomarkers; **(B)** Conducting multivariate analysis for significant clinical characters and hematological biomarkers.

To further investigate the prognostic significance of various factors in POC, univariate and multivariate analyses were performed. Univariate analysis revealed that age (hazard ratio [HR] = 0.58, 95% confidence interval [CI]: 0.14–2.4, P = 0.012) and LIPI (HR = 2.5, 95% CI: 1.6–3.8, P < 0.01) were significantly associated with overall survival (OS) (**Figure 5A**). Subsequently, multivariate analysis identified age (HR = 0.52, 95% CI: 0.27–0.98, P = 0.84) and LIPI (HR = 2.4, 95% CI: 1.5–3.8, P < 0.01) as independent prognostic factors for POC (**Figure 5B**).

### 3.4 Construction and validation of LIPI-based nomogram

**Figure 6:**
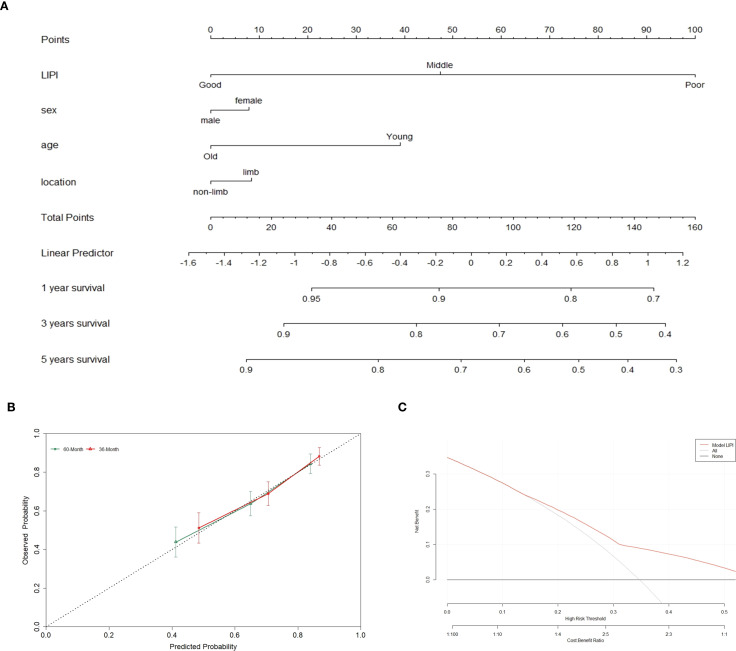
The overall survival nomogram of POC was constructed and validated. **(A)** LIPI, sex, age and location are combined to construct the nomogram, and the total score of the nomogram was the sum of the scores of each covariate. **(B, C)** The calibration curve and decision curve analysis verified the nomogram.

A nomogram integrating LIPI with clinical features was developed to improve its clinical applicability. Using the Cox proportional hazards regression model, scores were assigned based on the hazard ratios (HRs) of individual covariates, and the total nomogram score was obtained by summing these covariate scores (**Figure 6A**).

The calibration curve demonstrated that the nomogram effectively predicted 3- and 5-year overall survival (OS) in POC patients (**Figure 6B**). Furthermore, decision curve analysis (DCA) was employed to assess the clinical utility of the nomogram (**Figure 6C**). The results indicated that incorporating LIPI into the nomogram provided significant net benefits compared to a model based solely on clinical features.

## 4 Discussion

This retrospective study analyzed osteosarcoma patients who underwent chemotherapy to identify prognostic indicators associated with POC and to preliminarily validate the predictive utility of LIPI. The results demonstrated that LIPI serves as an independent risk factor for POC prognosis and exhibits superior prognostic accuracy compared to other hematological indices. Furthermore, a LIPI-based nomogram incorporating both LIPI and clinical features was successfully developed, enabling precise prediction of three- and five-year survival outcomes in POC patients. These findings suggest that LIPI may serve as a valuable and practical tool for prognostic assessment in POC.

Osteosarcoma remains the leading cause of tumor-associated mortality in adolescents and children (23). With advancements in comprehensive treatment, the OS rate has improved to 60%–70% for non-metastatic osteosarcoma patients (3). Despite advancements in treatment, significant heterogeneity in overall survival (OS) persists among osteosarcoma patients. Currently, traditional clinical factors, including the Enneking staging system, metastasis status, tumor site, histological type, and tumor grade, remain the primary prognostic indicators for osteosarcoma. (5). However, these factors have increasingly shown limitations, with discrepancies often observed between them and actual clinical outcomes (21). In recent years, several novel prognostic factors have been identified, including microRNAs, long non-coding RNAs (lnc-RNAs), and gene signatures, all of which have been reported to be effective in predicting osteosarcoma prognosis (6–10, 24). For example, our previous study demonstrated that a metabolic-related gene pair signature (MRGP) could reliably predict OS with an AUC of 0.9 in osteosarcoma patients (24). However, most of these biomarkers have not been validated in independent cohorts and are therefore not yet suitable for clinical application. Additionally, many of these biomarkers lack standardized detection methods, as the expression levels of miRNAs and lncRNAs can be influenced by variations in extraction and processing techniques. Consequently, inconsistencies in miRNA and lncRNA expression results are frequently reported. (25, 26). Most importantly, the high cost and inconvenience associated with detecting these biomarkers hinder their broader clinical use.

In contrast, hematological parameters derived from routine blood tests offer a low-cost, simple, and convenient approach to prognostic assessment. Numerous studies have demonstrated the prognostic significance of these biomarkers in cancer patients, with elevated levels of lactate dehydrogenase (LDH) and alkaline phosphatase (ALP) being associated with poor prognosis in osteosarcoma patients. (27–32).

Research has demonstrated significant correlations between inflammation and all stages of cancer development and malignant progression, as well as the effectiveness of anticancer therapies (33). Based on the Warburg effect, tumor cells exhibit increased glucose consumption and lactate production, which are key metabolic alterations during tumorigenesis and malignant transformation (34). LDH, a key enzyme in anaerobic glycolysis, is a well-recognized marker of poor prognosis in various cancers, including melanoma, osteosarcoma, and Ewing sarcoma (18, 35–37). Tumor-associated neutrophils (TANs) accumulate in specific tumor regions and can be activated by stimuli from the tumor microenvironment (TME), switching between anti-tumor and pro-tumor phenotypes (38). Several studies have shown that tumor-infiltrating lymphocytes can induce tumor cell apoptosis, influence immunotherapy responses, and release cytokines, playing crucial roles in mediating chemotherapy and immunotherapy responses (39–41). In our study, dNLR, which comprises neutrophils and lymphocytes, serves as an indicator of systemic inflammatory status in POC to some extent. Moreover, our findings, along with previous studies, suggest that the dNLR is a better prognostic predictor for POC than the NLR. This is because the dNLR includes additional inflammatory markers compared to the NLR, offering a more comprehensive reflection of tumor-related inflammation and thereby improving prognosis prediction (13, 18, 21). Similarly, Szkandera et al. reported a strong and independent correlation between high dNLR and poor OS in POC (42). Our study indicates that POC patients with an elevated serum dNLR (>1.59) tend to have a poorer prognosis (**Figure 3D**). Therefore, the LIPI, which integrates LDH and dNLR, may serve as a comprehensive marker of tumor-associated inflammation in POC, enabling more accurate prognostic assessments.

However, due to the complexity of the tumor microenvironment, a single hematological parameter is insufficient to comprehensively reflect an individual’s inflammatory status. Furthermore, the predictive capability of these individual biomarkers remains significantly inferior to that of metastasis status. Additionally, their predictive stability is limited, and their clinical significance varies across different studies, as observed with the lymphocyte-to-monocyte ratio (LMR). (7, 43). As awareness of the role of the inflammatory response in prognosis continues to grow, it is crucial to develop a comprehensive index that can accurately assess the inflammatory status and predict long-term survival. Several attempts have been made to integrate key inflammatory factors to better evaluate patient outcomes, such as the development of the LIPI in lung cancer (21).

Mezquita et al. introduced the LIPI, a comprehensive inflammation indicator calculated based on baseline dNLR and LDH, which aids in immunotherapeutic decisions and prognostication of OS in patients with advanced pulmonary and extrapulmonary malignancies (21, 22, 44). The efficacy of the LIPI in predicting prognosis has also been explored in various studies (20, 22). However, its prognostic value in POC remains unclear. Therefore, this study explored the correlation between LIPI, calculated using baseline LDH and dNLR, and POC, subsequently developing a LIPI-based prognostic model. Our findings indicate that LIPI outperforms individual hematological markers in predicting long-term survival in POC. Moreover, unlike single markers such as LDH, NLR, and dNLR, LIPI enables the stratification of patients into three distinct risk groups, thereby improving prognostic risk assessment and informing treatment decisions.

Time-dependent ROC curve analysis further demonstrated the superior prognostic performance of LIPI compared to other factors, highlighting its advantages over individual inflammatory markers. Additionally, the LIPI-based nomogram serves as a valuable tool for predicting OS in POC, facilitating the development of personalized treatment and follow-up strategies. For instance, patients with a high LIPI score may require more frequent follow-up visits and proactive interventions to improve long-term survival outcomes. By leveraging the LIPI-based nomogram, clinicians can implement tailored management strategies based on a patient’s prognostic risk.

However, our study has several limitations. First, as a single-center study, it may be subject to selection bias. Nevertheless, with 150 osteosarcoma patients following chemotherapy, this study is the first to specifically focus on POC. Given the sample size, our findings provide valuable insights into the role of LIPI in prognostic prediction for POC. Future research will include multicenter studies to further evaluate the efficacy of this predictive model.Second, the retrospective nature of this study introduces the potential for recall bias. However, conducting prospective studies remains challenging due to the rarity and heterogeneity of POC. To date, no prospective studies have investigated prognostic prediction in POC. Therefore, our future research will focus on multicenter, large-scale prospective studies to validate our findings and enhance their generalizability.

## 5 Conclusion

This study investigated the efficacy of the LIPI in predicting the prognosis of POC, categorizing patients into three groups to assess their prognosis. Additionally, a LIPI-based nomogram was developed to aid clinicians in predicting the three- and five-year OS of POC, potentially facilitating timely interventions and personalized management strategies.

## Data Availability

The raw data supporting the conclusions of this article will be made available by the authors, without undue reservation.
